# The effects on pain and quality of life of traditional Chinese manual therapy for knee osteoarthritis: A protocol for systematic review and meta-analysis

**DOI:** 10.1097/MD.0000000000028595

**Published:** 2022-01-21

**Authors:** Yu Zheng, Jun Ren, Shuaipan Zhang, Xin Zhou, Tianxiang He, Lingjun Kong

**Affiliations:** a Yueyang Hospital of Integrated Traditional Chinese and Western Medicine, Shanghai University of Traditional Chinese Medicine, Shanghai, China; b College of Acupuncture and Tuina, Shanghai University of Traditional Chinese Medicine, Shanghai, China; c Research Institute of Tuina, Shanghai Academy of Traditional Chinese Medicine, Shanghai, China.

**Keywords:** knee osteoarthritis, meta-analysis, quality of life, traditional Chinese manual therapy

## Abstract

**Background:**

Knee osteoarthritis (KOA) is a common disease with the high occurrence in the world. The symptoms of pain and dysfunction decrease quality of life in KOA patients. Several studies reported traditional Chinese manual therapy showed beneficial effects in improving pain and dysfunction of patient with KOA, but most previous reviews did not focus on the effects on quality life of traditional Chinese manual therapy for KOA. However, better quality of life is important for patients suffering KOA. Therefore, the current review and meta-analysis will be conducted to assess the effects on clinical symptoms and quality of life of traditional Chinese manual therapy for KOA.

**Methods:**

Eight electronic databases including PubMed, Embase, the Cochrane Library, Web of Science, Cochrane Central Register of Controlled Trials, China National Knowledge Infrastructure, Wanfang Data, and Chinese Scientific Journal Database will be searched from the beginning to December 2021. Two reviewers will independently select included studies and extract data. Heterogeneity will be evaluated by *I*^*2*^ statistic before the data synthesis. Subgroup analysis will be performed by duration of KOA, different types of traditional Chinese manual therapy, different outcomes, and different intervention time. The primary outcome is quality of life in KOA patients, and the secondary outcomes include pain and dysfunction due to KOA. Rev Man 5.3 software will be used for meta-analysis.

**Results:**

The results of this review will be reported in a peer-reviewed journal.

**Conclusion:**

The results of this review will provide reliable evidence for the effects on quality of life and clinical symptoms of traditional Chinese manual therapy for KOA.

**INPLASY registration number:**

INPLASY2021120043

## 1 Introduction

Knee osteoarthritis (KOA) is a common disease with prevalence rising with age, prevalence peaks at around 50 years of age,^[1]^ occurs in 10% to 30% older people and caused a serious burden.^[2]^ In the last 20 years, the KOA incidence was doubled in women and tripled in men.^[3]^ Potential long-term consequence of KOA included reducing physical activity, impaired sleep, fatigue, depression, functional decline even disability, it decreased quality of life.^[4–6]^ Risk factors include old age, diet, obesity, sedentary lifestyle, knee injury, pain, and frailty.^[7,8]^ In United States, about 10% adults suffer from KOA and have poor quality of life,^[9]^ the physical and psychosocial impairments in individuals with KOA have an impact on quality of life due to their influence on social interactions, pain, functional limitations, and sleep quality.^[10–13]^

Effective management of KOA requires long-term treatment strategies for clinical symptoms and joint structure changes that lead to disability.^[14]^ Standard treatments of KOA include drug therapy, joint injections, assistive devices, physical therapy, exercise, orthopedic aids, orthoses, and joint replacement surgery.^[9,15]^ Each treatment provides some benefits, but many KOA patients continue to suffer pain and functional limitations. Furthermore, they have led to undesirable side effects including heart failure, hypertension, etc,^[16–18]^ lead to compromised quality of life, even when utilizing multiple therapies.

In recent years, owing to the limitations of pharmacotherapy and the evidence that nonpharmacologic treatment are more likely to improve pain and dysfunction in the long term, there is a shift from primarily pharmacologic therapy to nonpharmacologic therapy, including physical therapy and exercise.^[4,19]^ Traditional Chinese manual therapy as a complementary and alternative treatment for KOA has a history of more than 2000 years in China.^[20]^ Previous studies have carried out in traditional Chinese manual therapy to prove its effects for KOA.^[20,21]^ But the effects of traditional Chinese manual therapy for KOA remain controversial. Most previous reviews did not focus on quality of life of traditional Chinese manual therapy for KOA. Therefore, this review will assess the evidence on quality of life and clinical symptoms of traditional Chinese manual therapy for KOA.

## 2 Methods

### 2.1 Study registration

The current protocol of systematic review has been drafted based on the preferred reporting items for systematic reviews and meta-analyses protocols statement guidelines,^[22]^ was registered with the International Platform of Registered Systematic Review and Meta-Analysis Protocols on December 8, 2021. The registration number is INPLASY2021120043.

### 2.2 Inclusion criteria

#### 2.2.1 Types of studies

Randomized controlled trials of traditional Chinese manual therapy for KOA will be included in this review, whether or not the expression “randomization” is mentioned with the randomization methods. Other studies including case report, theoretical or basic research, retrospective studies will be excluded.

#### 2.2.2 Types of participants

In this review, patients with KOA^[23]^ will be included regardless of sex, age, race, or duration of disease.

#### 2.2.3 Types of interventions

Studies applied traditional Chinese manual therapy in the experimental group will be included. The control group will include drug therapy, joint injections, assistive devices, physical therapy, exercise, orthopedic aids, acupuncture, and other therapies without manual therapy.

### 2.3 Types of outcome measures

#### 2.3.1 Primary outcome

The quality of life will be measured by the 36-item Short Form Health Survey.^[24]^

#### 2.3.2 Secondary outcomes

The pain will be measured by visual analog scale.^[25]^ The functional outcomes will be measured by the Western Ontario and McMaster Universities Osteoarthritis Index.^[26]^

### 2.4 Data sources

The following databases will be searched, including Embase, PubMed, Web of Science, Cochrane Library, Cochrane Central Register of Controlled Trials, China National Knowledge Infrastructure, Wanfang Data, and Chinese Scientific Journal Database. We will search electronic databases from the beginning to December 2021. The search strategy for PubMed is shown in Table 1.

**Table 1:** Search strategy for PubMed database.

No.	Search items
#1	“traditional Chinese manual therapy” [tiab] or “Chinese massage” [tiab] or “Tuina” [tiab] or “Chinese spinal manipulation” [tiab]
#2	“Knee osteoarthritis” [tiab] or “Osteoarthritis of knee” [tiab] or “Knee osteoarthritides ”[tiab] or “Senile osteoarthritis” [tiab] or “Genual osteoarthritis” [tiab] or “Osteoarthritis” [tiab] or “Arthritis” [tiab] or “Degenerative arthritis” [tiab] or “Hypertrophic arthritis” [tiab] or “Gonarthrosis” [tiab]
#3	“Randomized controlled trial” [article type]
#4	#1 and #2 and #3

### 2.5 Search strategy

#### 2.5.1 Study selection

**Figure 1:**
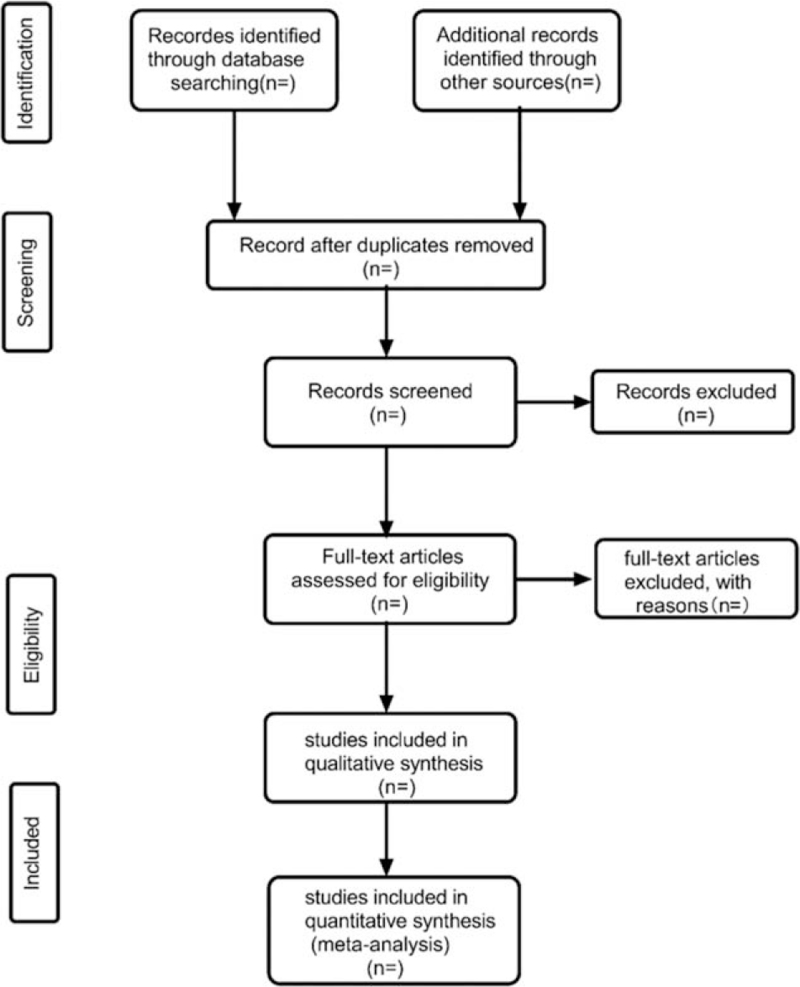
Flow diagram of studies selection process.

Two reviewers will independently select literatures by reading the titles and abstracts according to the eligibility criteria that have been discussed by all reviewers. The full-text of potential studies will be reed for final included studies. Any disagreements will be resolved through discussion and consensus between researchers. The entire process of the studies selection will be summarized in a flow diagram with Figure 1.

#### 2.5.2 Data extraction

Based on discussion and consensus, 2 researchers will independently extract data from the included studies including basic information (the first author, year of publication, and country), participant information (age, gender, diagnostic criteria, and duration of KOA), interventions information (type of traditional Chinese manual therapy, time of treatment, frequency of treatment, duration, and follow-up), and outcomes information (the primary outcome is quality of life; the second outcomes are pain, and dysfunction), and other project data (funding sources and ethical approvals). Any disagreements will be resolved through discussion and consensus between researchers.

### 2.6 Quality assessment

Two reviewers will independently assess the quality assessment for included trials according to Cochrane tool with risk of bias, including random sequence generation, allocation concealment, blinding of patients, blinding of testers, blinding of outcome evaluators, incomplete outcome data, and selective reporting. The quality of evidence will be evaluated by Grades of Recommendation, Assessment, Development, and Evaluation framework including the risk of bias, inconsistency, indirectness, imprecision, and publications bias. Any disagreements will be resolved through discussion and consensus among researchers.

### 2.7 Data synthesis and analysis

The Review Manager Version 5.3 software (The Nordic Cochrane Centre, Copenhagen, Denmark) will be used in the meta-analysis. For continuous data, the standardized mean difference and 95% confidence interval will be used. The dichotomous data will be presented as the risk ratio values and the corresponding 95% confidence interval.

### 2.8 Heterogeneity analysis

Heterogeneity will be assessed by the test of *I*^2^ before the data synthesis. The random effect model (*I*^2^ ≥ 50%) or fixed-effect model (*I*^2^ < 50%) will be used in the meta-analysis.

### 2.9 Subgroup analysis

Subgroup analysis will be conducted based on duration of KOA, different type of traditional Chinese manual therapy, different outcomes, and different intervention time.

### 2.10 Sensitivity analysis

Sensitivity analysis will be performed to assess the robustness and reliability of the combined results in the meta-analysis. This evaluation refers to sensitivity analyses.^[27]^ An Egger test will be used to assess publication bias of the included studies.

### 2.11 Ethics and dissemination

The systematic review does not require ethical approval because there are no data used in the trials that are linked to individual patient data. In addition, the results of the current review will be published in a peer-reviewed journal.

## 3 Discussion

KOA is the most common disease with rank highly among global causes of disability and chronic pain.^[1]^ The surgical procedures and alternative treatments both focus on improving quality of life in KOA patients. For early KOA, complementary and alternative therapy is usually the first option, especially for KOA without clear lesions or associated abnormalities.^[28]^ Traditional Chinese manual therapy is one of complementary and alternative therapies, that is with a high safety when used by trained therapists.^[9]^ The study has reported that traditional Chinese manual therapy improved pain, edema, and weakness of muscles of KOA.^[29]^ It may be benefit from improving muscle tone, blood circulation, flexibility, and inhibition of inflammatory factors.^[30]^ However, there is a lack of systematic reviews on quality of life of traditional Chinese manual therapy for KOA. Therefore, the current systematic review and meta-analysis will be conducted to assess the evidence on quality of life and clinical symptoms of traditional Chinese manual therapy for KOA. The findings will provide the value option for clinicians and patients to use traditional Chinese manual therapy for KOA.

## Author contributions

**Conceptualization:** Yu Zheng, Jun Ren, Shuaipan Zhang.

**Funding acquisition:** Tianxiang He, Lingjun Kong.

**Methodology:** Yu Zheng, Shuaipan Zhang, Xin Zhou.

**Project administration:** Jun Ren, Shuaipan Zhang, Tianxiang He.

**Writing – original draft:** Yu Zheng, Jun Ren, Shuaipan Zhang, Xin Zhou.

**Writing – review & editing:** Tianxiang He, Lingjun Kong.
